# The increasing impact of cerebral amyloid angiopathy: essential new insights for clinical practice

**DOI:** 10.1136/jnnp-2016-314697

**Published:** 2017-08-26

**Authors:** Gargi Banerjee, Roxana Carare, Charlotte Cordonnier, Steven M Greenberg, Julie A Schneider, Eric E Smith, Mark van Buchem, Jeroen van der Grond, Marcel M Verbeek, David J Werring

**Affiliations:** 1 Stroke Research Centre, Department of Brain Repair and Rehabilitation, UCL Institute of Neurology and the National Hospital for Neurology and Neurosurgery, London, UK; 2 Division of Clinical Neurosciences, Faculty of Medicine, University of Southampton, Southampton, UK; 3 Department of Neurology, Université de Lille, Inserm U1171, Degenerative and Vascular Cognitive Disorders, Centre Hospitalier Régional Universitaire de Lille, Lille, France; 4 J P Kistler Stroke Research Center, Massachusetts General Hospital, Harvard Medical School, Boston, MA, USA; 5 Departments of Pathology and Neurological Sciences, Rush Alzheimer’s Disease Center, Rush University Medical Center, Chicago, IL, USA; 6 Hotchkiss Brain Institute, Department of Clinical Neurosciences, University of Calgary, Calgary, Alberta, Canada; 7 Department of Radiology, Leiden University Medical Center, Leiden, The Netherlands; 8 Radboud University Medical Center, Donders Institute for Brain, Cognition and Behaviour, Nijmegen, The Netherlands; 9 Departments of Neurology and Laboratory Medicine, Radboud Alzheimer Center, Nijmegen, The Netherlands

**Keywords:** cerebrovascular disease, amyloid, stroke, vascular dementia, superficial siderosis

## Abstract

Cerebral amyloid angiopathy (CAA) has never been more relevant. The last 5 years have seen a rapid increase in publications and research in the field, with the development of new biomarkers for the disease, thanks to advances in MRI, amyloid positron emission tomography and cerebrospinal fluid biomarker analysis. The inadvertent development of CAA-like pathology in patients treated with amyloid-beta immunotherapy for Alzheimer’s disease has highlighted the importance of establishing how and why CAA develops; without this information, the use of these treatments may be unnecessarily restricted. Our understanding of the clinical and radiological spectrum of CAA has continued to evolve, and there are new insights into the independent impact that CAA has on cognition in the context of ageing and intracerebral haemorrhage, as well as in Alzheimer’s and other dementias. While the association between CAA and lobar intracerebral haemorrhage (with its high recurrence risk) is now well recognised, a number of management dilemmas remain, particularly when considering the use of antithrombotics, anticoagulants and statins. The Boston criteria for CAA, in use in one form or another for the last 20 years, are now being reviewed to reflect these new wide-ranging clinical and radiological findings. This review aims to provide a 5-year update on these recent advances, as well as a look towards future directions for CAA research and clinical practice.

## Introduction

Cerebral amyloid angiopathy (CAA), a cerebral small vessel disease (SVD) characterised by the presence of amyloid-beta (Aβ) protein within cortical and leptomeningeal blood vessel walls,^1^ is a condition of increasing clinical and mechanistic importance. Although recognised pathologically since the early 20th century, CAA had been sidelined until it was more firmly associated with lobar intracerebral haemorrhage (ICH) many years later.^2 3^ This, together with the use of blood-sensitive magnetic resonance sequences to visualise asymptomatic haemorrhagic events that far exceed those causing clinical symptoms, has expanded our understanding of what CAA is and its clinical significance.^2–4^ The last 5 years have seen ongoing progress in this area, thanks in part to the development of new technologies within the fields of magnetic resonance, amyloid positron emission tomography ligands and cerebrospinal fluid (CSF) biomarker analysis.^4^ Furthermore, CAA gained new relevance with the advent of anti-Aβ immunotherapies for the treatment of Alzheimer’s disease (AD), as a sizeable proportion of those treated went on to develop imaging features of CAA-related inflammation as an unintended consequence.^5^ This, together with advances in our understanding of the impact of CAA on cognition, in the context of ICH, ageing and AD, has broadened the clinical spectrum of disease to which the contribution of CAA is recognised. The 5th International CAA Conference, held in Boston, Massachusetts in September 2016 (https://caaforum.org/event/5th-international-caa-conference-2016-boston-ma/), highlighted many of these developments, as well as a need to update the current diagnostic Boston criteria to better reflect these new wide-ranging clinical and radiological findings.

This review aims to provide a clinically oriented update on these recent advances, focusing on those in the last 5 years, since our last comprehensive review.^3^ Specifically, we will focus on newly identified biomarkers for CAA and their potential mechanistic implications, as well as the latest insights into the pathophysiology and causes of CAA. We will also report on the expanding spectrum of clinical presentations associated with CAA, before finally considering the ongoing management dilemmas that face physicians working in neurology, elderly care and stroke medicine, among others, when faced with this disease.

## Biomarkers advances and mechanistic insights

### Insights from hereditary cerebral haemorrhage with amyloidosis-Dutch type (HCHWA-D)

Establishing the natural history of sporadic CAA is difficult, in part due to the limited sensitivity of our current diagnostic criteria,^6 7^ and also due to the high prevalence of comorbid changes caused by ageing and other neurodegenerative disorders. Moreover, most of these clinical and radiological features are thought to occur  relatively late in the disease process; given that any potential window for treatment is likely to be at earlier stages of the disease, this is likely to significantly limit their use as feasible biomarkers.

HCHWA-D is an autosomal dominant disease that predominantly occurs in a limited number of families in the villages of Katwijk and Scheveningen in the Netherlands.^8^ A point mutation (E693Q) of the amyloid precursor protein (APP) gene^9^ leads to extensive Aβ deposition in the cortical and leptomeningeal arterioles and arteries. The underlying pathology of these Aβ deposits is similar to that in sporadic CAA with minimal or no neurofibrillary pathology.^8 9^ Most importantly, mutation carriers develop symptoms (recurrent ICH and dementia^10^) relatively early in life, usually between the ages of 50 and 60 years. Therefore, using HCHWA-D as a model allows CAA-related changes to be studied with minimal confounding by comorbidities associated with ageing. This genetic condition also opens the possibility to find new disease markers, even in the presymptomatic phase.

**Figure 1:**
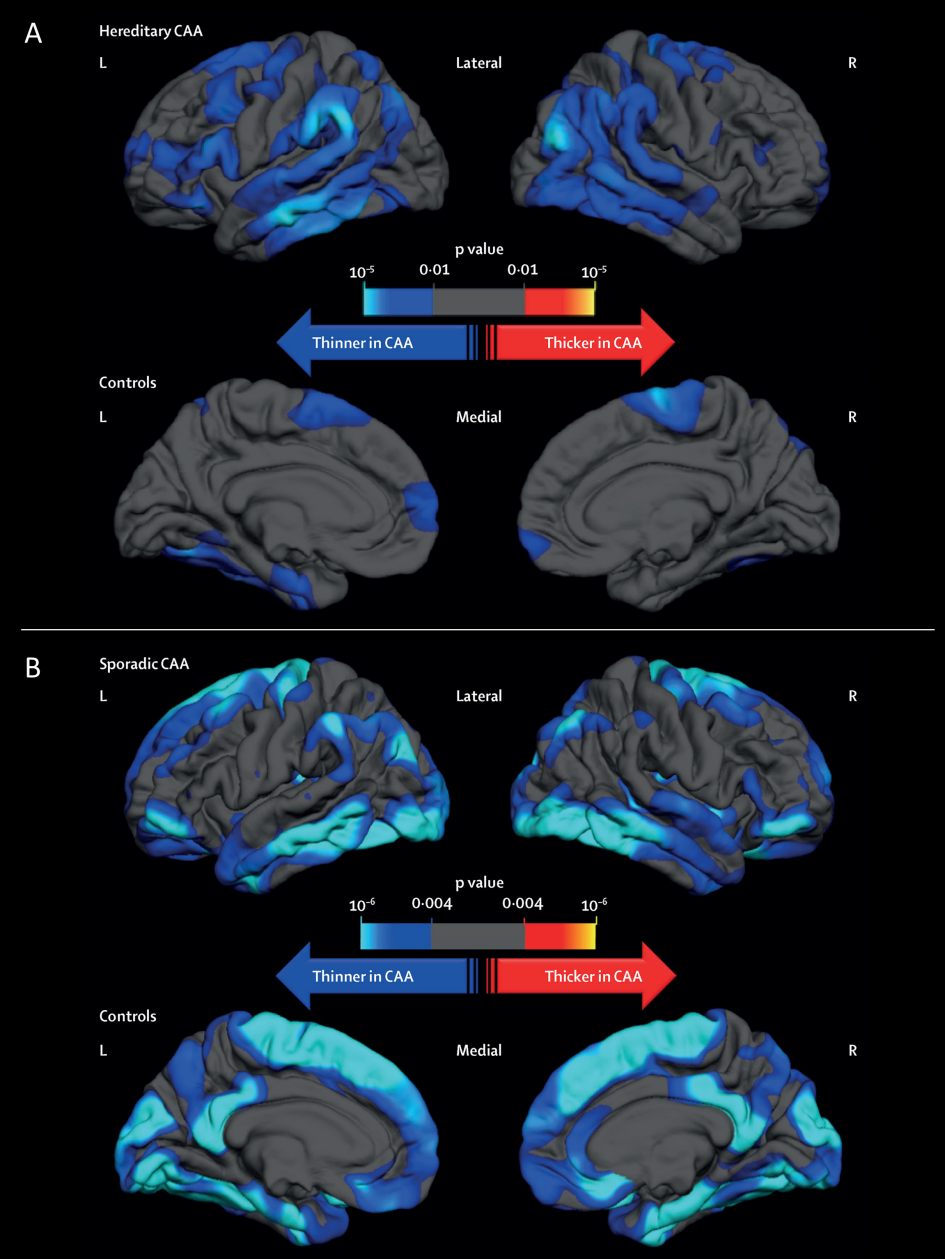
Differences in cortical thickness between patients with (A) hereditary cerebral haemorrhage with amyloidosis–Dutch type and (B) sporadic cerebral amyloid angiopathy, and their respective age-matched controls. A general linear model was computed to schematically explore the regional differences in cortical thickness between patients with (A) HCHWA-D and healthy controls and (B) sporadic CAA and healthy controls, after adjusting for age and sex. Topographic surface maps were generated using a threshold of p<0.01 (with false discovery rate correction for multiple comparisons). The resulting maps show the statistically significant regional differences in cortical thickness. CAA, cerebral amyloid angiopathy; HCHWA-D, hereditary cerebral haemorrhage with amyloidosis–Dutch type; L, left; R, right. These panels have been reproduced without modification from^14^ (DOI: 10.1016/S1474-4422(16)30030-8), under the terms of the Creative Commons Attribution-NonCommercial-No Derivatives License (CC BY NC ND; https://creativecommons.org/licenses/by-nc-nd/4.0/legalcode).

Previous studies have shown that the characteristic radiological signs of sporadic CAA are mimicked in symptomatic HCHWA-D, namely numerous lobar microbleeds with or without cortical superficial siderosis (cSS).^3 11 12^ In presymptomatic HCHWA-D, haemorrhagic changes are present, but more subtle; in recent studies, none or only one of the ‘classical’ signs (lobar microbleeds, macrohaemorrhage, cSS or convexity subarachnoid haemorrhage, cSAH) were present.^13^ However, other disease markers such as white matter hyperintensities (WMH) and cortical microinfarcts, which are ischaemic manifestations of CAA,^3^ were  already prevalent among presymptomatic HCHWA-D subjects.^13^ This indicates that these disease manifestations are among the earliest markers of the hereditary form of CAA and precede ICH. In addition to explicit white matter changes, the grey matter is also specifically but subtly affected; recent data clearly showed that both patients with CAA and HCHWA-D demonstrate similar patterns of cortical thinning, suggesting that vascular Aβ is an independent contributor to cortical atrophy (figure 1).^14^

Vascular reactivity is measured by blood-oxygen level-dependent (BOLD) amplitude, time to response and time to return to baseline after visual stimulation. Cerebrovascular dysfunction is intrinsically associated with CAA disease pathology by Aβ deposition in the vessel wall, resulting in impaired vascular reactivity in symptomatic patients with CAA.^15^ Recently it was shown that similar changes are also present in symptomatic HCHWA-D.^16^ Determination of vascular reactivity in symptomatic and presymptomatic HCHWA-D showed that in both groups vascular reactivity was significantly decreased in the occipital lobe.^16^ Although this effect was far more pronounced in the symptomatic group, raising the possibility that these effects may be influenced by the presence of occipital macrohaemorrhage, the finding of reduced vascular reactivity even in presymptomatic patients with HCHWA-D suggests it as an early step in CAA pathogenesis.

Both the haemorrhagic and haemodynamic cerebral changes are secondary effects of Aβ deposition in the cerebrovascular vessel wall^17^; a key future goal is to determine the level of Aβ deposition in the vessel wall itself. Measurement of Aβ species in the CSF shows reductions in both Aβ40 and Aβ42 in presymptomatic as well as symptomatic patients with HCHWA-D.^18^ These findings point to Aß deposition as another marker of the earliest phase of CAA-related pathology, occurring before clinical or radiological findings appear.

In summary HCHWA-D appears to be an excellent model to study important aspects of CAA, particularly presymptomatic brain imaging changes, which are much more difficult to investigate in sporadic CAA. With further biomarker development, HCHWA-D might also have promise for testing potential new disease-modifying therapies with potential relevance for sporadic CAA.

### Related developments in sporadic CAA

#### Imaging

Neuroimaging remains a central component to the diagnosis of CAA, with a focus on the presence of the haemorrhagic manifestations of the disease: lobar macrohaemorrhage, strictly lobar microbleeds and cSS.^6 7^ The last 5 years have seen increased understanding of these markers, as well as the emergence of new potential structural and functional imaging markers for CAA, all of which may be of both diagnostic and mechanistic relevance. Some notable recent advances are summarised here (table 1).^19–30^

**Table 1:** Summary of potential new structural and functional imaging markers for CAA

Imaging marker	Evidence of potential as a biomarker in CAA	Limitations
MRI visible perivascular spaces in the centrum semiovale (CSO-PVS)	Severe or high-grade CSO-PVS commonly observed in CAA^19–22^ Pilot data show that, in those with CAA, CSO-PVS severity is associated with Aβ burden (as measured by PiB)^23^	Non-specific (age-related); present in a number of other conditions^24^
Cortical atrophy	Thinner cortices in those with sporadic CAA compared with healthy controls; occipital, temporal, posterior parietal and medial frontal areas affected^14^ (figure 1)	Difficult to differentiate between atrophy secondary to parenchymal Aβ and that due to vascular Aβ in sporadic CAA
Visual functional MRI	Patients with CAA have abnormal BOLD responses to a visual stimulus (alternating checkerboard), with reduced response amplitude and prolonged time both to peak and to baseline^15 25^ Those with CAA show a decline in BOLD amplitude that is detectable at 1 year; longitudinal difference in BOLD amplitudes was significantly lower in CAA compared with controls^26^ Potentially of interest as a surrogate marker of vascular health in clinical trials	Clinical implications of this remain unclear; due to technical factors at this stage, this is predominantly a research tool limited to academic medical centres
Network measures	Lower global efficiency of brain network in those with CAA; occipital, parietal and posterior temporal lobes most affected^103^ Reduced efficiency correlated with Aβ burden (as measured by PiB) and impaired executive function and processing speed^103^ Global efficiency shows a longitudinal decline with time (1.3 years) in those with CAA, and is associated with deteriorating executive function^104^	Difficult to differentiate between network effects of parenchymal Aβ versus those due to vascular Aβ in sporadic CAA Mainly a research tool limited to academic medical centres
Amyloid PET imaging using [^11^C] PiB-PET and [^18^F] compounds	In those with CAA, regions with high PiB retention area associated with subsequent haemorrhage^27^ Although PiB-PET may not reliably distinguish between patients and age-matched controls,^28^ early phase (1–6 min) uptake can do this^29^ The occipital/posterior cingulate ratio of PiB uptake is different for those with CAA versus those with AD^29^ PiB-PET and [^18^F] florbetapir binding is able to distinguish between CAA-associated ICH and hypertension-associated ICH^30^	Amyloid PET unable to differentiate between vascular and parenchymal Aβ Diagnostic accuracy for CAA seems limited Few data on change over time in CAA

Aβ, amyloid-beta; AD, Alzheimer’s disease; BOLD, blood-oxygen level-dependent; CAA, cerebral amyloid angiopathy; ICH, intracerebral haemorrhage; PET, positron emission tomography; PiB, Pittsburgh B compound.

While multiple lobar ICH remains the strongest indicator for CAA, they are a late feature of the disease and may not be a practical outcome marker for clinical trials.^31^ The association between cSS and CAA is now well recognised, reflected by its inclusion in the modified Boston criteria.^7^ Clinically, cSS is associated with transient focal neurological episodes (TFNE)^32^ and an increased risk of ICH,^33–35^ including early recurrent ICH.^36^ There is also increasing evidence that cSS is associated with cognitive impairment, although it is not clear whether this is because cSS is a marker of severe CAA or because it has direct and independent effects on cognition.^37–45^ The other haemorrhagic marker of CAA, the presence of strictly lobar microbleeds, remains central to diagnosis. However, recent work reviewing diagnostic accuracy of using strictly lobar microbleeds alone found that while they were strongly predictive of CAA in a hospital cohort, this was not the case in a healthy community population.^46^ This has highlighted the importance of identifying new imaging markers for CAA that can further improve the specificity and sensitivity of diagnosis,^31 47^ particularly outside ICH cohorts.

In the last 5 years new structural and functional imaging markers for CAA have emerged. As highlighted in a recent review,^31^ identifying new markers that could be used in therapeutic clinical trials is an urgent priority for CAA. Establishing CAA severity using neuroimaging also remains a challenge; a composite score has been proposed,^48^ which aims to estimate the overall pathological ‘burden’ of CAA in a given patient by combining key imaging markers of CAA, with some preliminary pathological verification of the concept. Evaluating this score against meaningful clinical outcomes will be essential for establishing whether it adds to the predictive value of individual markers or has practical application in clinical practice or future trials.

#### Body fluid biomarkers in sporadic CAA

Since CAA may arise due to the impaired clearance of Aβ from interstitial cerebral fluid, CSF should contain biomarkers that reflect this process. A key factor in understanding the significance of the biomarkers in CSF will be to identify exactly the pathways for clearance of peptides from the interstitial fluid into the CSF.

It has been demonstrated that CAA-affected vessels, unlike senile plaques, contain significant Aβ40 in addition to Aβ42,^49^ which is abundantly found in senile plaques. This specific pattern of Aβ40/42 deposition in CAA is reflected by decreased levels of both Aβ40 and Aβ42 peptides in CSF obtained from patients with CAA, diagnosed according to the Boston criteria,^50^ a  finding replicated by other groups.^51^ Similarly, decreased CSF Aβ40 and Aβ42 concentrations were observed in patients with isolated cSS.^52^ A rare variant of CAA is CAA-related inflammation (CAA-ri), also known as Aβ-related angiitis. Patients with this condition suffer from acute or subacute neurological impairment, often including headache, encephalopathy, behavioural changes, seizures and focal neurological deficits. During the acute phase of CAA-ri, increased levels of anti-Aβ autoantibodies can be found in the CSF,^53^ as well as decreased Aβ40 and Aβ42 levels.^54^

In single case reports of CAA caused by the Dutch-type Aβ E22Q mutation and the Iowa-type Aβ D23N mutation, similarly decreased levels of Aβ40 and Aβ42 peptides were found in CSF^50^; the results of larger studies suggest that these peptides might be promising preclinical markers of CAA, as discussed in the preceding section on HCHWA-D.^18^ Decreasing CSF Aβ40 concentrations in these patients were associated with higher lobar microbleed count, increasing WMH volume and presence of cSS, and in addition were visible prior to the abnormalities present on neuroimaging.

As expected, levels of both total and phosphorylated tau protein are not specifically related to CAA and were marginally elevated in CSF from patients with CAA compared with controls, but were substantially lower than in patients with AD.^50 51 54^ Total tau protein levels are increased in CAA-ri, but both normal and increased levels of phosphorylated tau proteins have been described.^51 53 54^ These minor elevations in CSF tau protein levels are probably attributable to a low level of concomitant AD pathology (neurofibrillary tangles) in patients with CAA. In contrast to the observations in sporadic CAA, both CSF total tau and phosphorylated tau concentrations were decreased in symptomatic, but not in presymptomatic patients with HCHWA-D compared with controls, although the difference in total tau disappeared after correction for age.^18^

Given the association of CAA with AD, CSF Aβ protein levels have also been studied in correlation with the presence of CAA in patients with dementia, in which strictly lobar microbleeds on MRI (T2* or susceptibility weighted imaging, SWI) were used as the indicator of CAA. An independent correlation between decreased CSF Aβ42 and the presence of cortical microbleeds was demonstrated in a heterogeneous cohort of patients with dementia.^55^ Patients with AD and with multiple microbleeds (defined as more than 8) also had lower CSF Aβ42 levels, compared with patients with AD without microbleeds,^56^ but such differences were not found for CSF Aβ40 levels.^56–58^ This is in contrast with recent data that showed reductions in both CSF Aβ40 and Aβ42 in patients with AD and strictly lobar microbleeds compared with those with AD without microbleeds.^59^

By contrast with CSF biomarkers, studies on the association between blood levels of Aβ proteins and the presence of CAA are rather limited. In patients with sporadic CAA, plasma Aβ40, but not Aβ42, concentrations were associated with WMH,^60^ indicating that circulating Aβ peptides might be an indicator of cerebral microvascular damage. Moreover, in patients with multiple CAA-related ICH, both plasma Aβ42 and Aβ40 concentrations were higher than in controls.^61^ A single study described that plasma Aβ42, but not Aβ40, levels were decreased in patients with HCHWA-D.^10^

Together, these data highlight the promise that body fluid biomarkers hold as potential preclinical markers for CAA. Further work, in particular in identifying viable plasma biomarkers, is needed.

## The pathophysiology of vascular Aβ deposition: an update

### Perivascular Aβ clearance

The deposition of Aβ in the basement membranes of cerebral capillaries and arteries maps the intramural perivascular drainage pathways.^62 63^ When physiological small volumes of Aβ40 or other solutes are injected into the grey matter of experimental rodent brains, the Aβ40 enters basement membranes of capillaries and basement membranes surrounding smooth muscle cells of arteries within 5 min of intracerebral injection, mirroring the deposition of Aβ in CAA.^64^ Vascular basement membranes are specialised forms of extracellular matrix, composed of glycoproteins such as collagen IV and laminin, as well as proteoglycans such as agrin and perlecan. With increasing age and with possession of ApoE ε4 genotype, vascular basement membranes change their composition, with reduced functional efficiency.^65 66^ The perivascular drainage of solutes is thus impaired in ageing and with possession of ApoE ε4, further adding to the burden of failure of other clearance mechanisms.^65–68^

Indeed, apart from intramural perivascular drainage, other mechanisms of breakdown and clearance of Aβ from the brain include enzymatic breakdown, clearance via the low-density lipoprotein receptor-related protein 1 (through which most of Aβ is cleared), and phagocytosis by perivascular macrophages, astrocytes or microglia.^69–72^ Possession of ApoE ε4 and ageing impact adversely on all these molecular and cellular clearance mechanisms, as well as on ATP-binding cassette transporters involved in the clearance of Aβ.^73–75^

**Figure 2:**
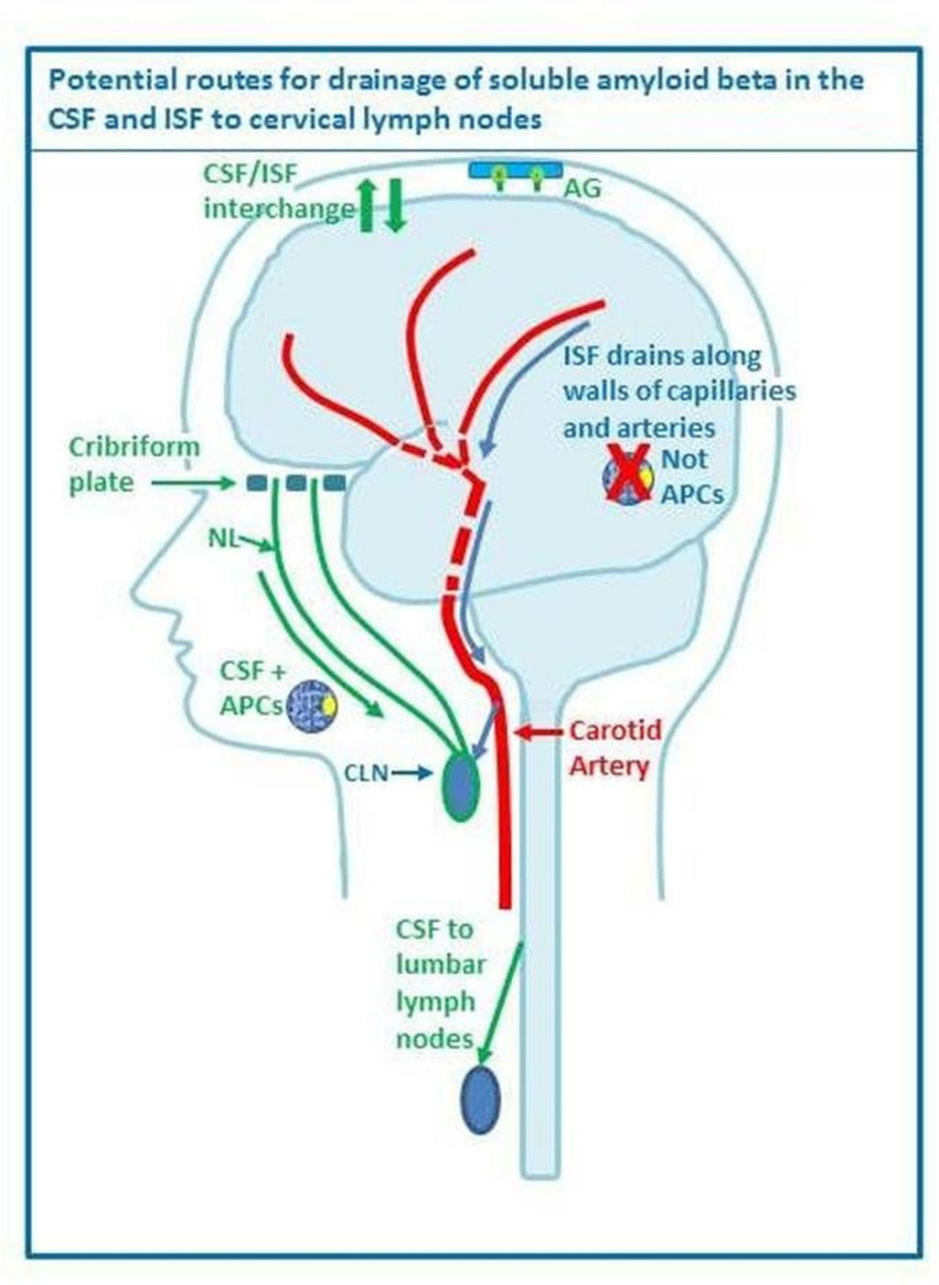
Drainage pathways for CSF and interstitial fluid (ISF) to cervical lymph nodes. With permission from Engelhardt *et al*.^81^ CSF and ISF drain to lymph nodes by different and distinct pathways. In humans, CSF drains into the blood of venous sinuses through well-developed arachnoid villi and granulations (AG). Lymphatic drainage of CSF occurs via nasal and dural lymphatics and along cranial and spinal nerve roots (outlined in green). Channels that pass from the subarachnoid space through the cribriform plate allow passage of CSF (green line) T cells and antigen presenting cells (APC) into nasal lymphatics (NL) and cervical lymph nodes (CLN). CSF from the lumbar subarachnoid space drains to lumbar lymph nodes. ISF from the brain parenchyma drains along basement membranes in the walls of cerebral capillaries and arteries (blue arrows) to cervical lymph nodes adjacent to the internal carotid artery just below the base of the skull. There is interchange between CSF and ISF (convective influx/glymphatic system) as CSF enters the surface of the brain alongside penetrating arteries.

The convective influx of CSF into the brain parenchyma has also received attention recently in relation to the clearance of Aβ.^76^ When horseradish peroxidase or fluorescent Aβ are injected into the CSF, they are observed in the walls of both arteries and veins at 30 min after injection into the CSF.^77 78^ As this process depends on optimal expression of aquaporin 4 forming channels present in the astrocyte end feet—and regulating water exchange—it has been named ‘glymphatic’ drainage. On a closer look using nanoparticles injected into the CSF and electron microscopy, the pial-glial basement membranes of arteries have been defined as the pathways for convective influx—or glymphatic drainage—of CSF into the cerebral parenchyma, with nanoparticles entering the brain within 5 min of their injection into the CSF compartment.^79^ It is difficult to interpret the relevance of this pathway in the context of pathogenesis of CAA, as Aβ is produced within the parenchyma and drains towards the leptomeningeal surface of the brain. The interplay between intramural drainage of interstitial fluid from the parenchyma along the basement membranes surrounding smooth muscle cells and the convective influx (or glymphatic drainage) of CSF along the pial-glial basement membranes into the parenchyma thus remains to be investigated further, as it probably plays a key role in the maintenance of homeostasis of the brain and in the development of diseases of Aβ accumulation^80^ (figure 2).^81^

### Amyloid-related imaging abnormalities (ARIA): friend or foe?

Further insights into how and why CAA may occur have come from the recent immunotherapy trials for AD. All such trials have reported ‘Amyloid Related Imaging Abnormalities’ (ARIA) as an unintended adverse effect; these are defined by their neuroimaging appearances as either predominantly oedematous or haemorrhagic, with the former showing vasogenic oedema and the latter developing cerebral microbleeds and cSS.^5^ ARIA shares a striking similarity of imaging features with the rare inflammatory variant of CAA (CAA-ri) in which spontaneously generated antibodies against vascular Aβ are observed.^53 82^ This apparent antibody-mediated development of imaging features found in CAA raises the possibilities that CAA may either develop as a consequence of rapid removal of parenchymal Aβ or trigger ARIA if already present when exposed to anti-Aβ antibodies. Higher and more effective doses of Aβ immunotherapy are more likely to result in ARIA,^5 83 84^ and areas affected by ARIA are associated with subsequent parenchymal Aβ clearance.^5 85 86^ The persistence of CAA after immunisation against Aβ42 suggests that the solubilised Aβ from plaques becomes entrapped in the intramural periarterial drainage pathways. Recent neuropathological work found that sites of microhaemorrhage are associated with lower levels of vascular Aβ and less severe CAA,^87^ which again may suggest that the neuroimaging findings seen in ARIA and associated with CAA might be manifestations of attempted Aβ clearance. The pathophysiological process by which parenchymal Aβ shifts to the vasculature may be mediated by ApoE, which is involved both in Aβ transfer across the blood–brain barrier and intramural periarterial Aβ transport^85^; this could explain the increased incidence of ARIA events in those with the ApoE ε4 allele.^88^ Aβ immunotherapy seems to result in a shift in ApoE localisation that mirrors Aβ movement, with reductions in plaque-associated ApoE and an increase in cerebrovascular expression.^85^ However, the clinical relevance of ARIA remains unclear, since in one recent small immunotherapy trial the biggest clinical improvement was seen in those with ARIA,^83^ suggesting that ARIA may in fact be a marker of efficacy of amyloid clearance, rather than only a deleterious side effect. Furthermore, ARIA often causes few clinical symptoms in comparison to CAA-ri.^5^ Thus it is possible that, while inflammatory CAA and ARIA are manifestations of active Aβ clearance, sporadic CAA represents a quiescent period of disease, evidence of either previous parenchymal clearance or ongoing low-level clearance, which like many cases of ARIA may be asymptomatic. Better understanding of these processes is essential, for extending the future therapeutic use of Aβ immunotherapies and for understanding the mechanisms by which ICH occurs in CAA.

## Clinical impact on cognition

### CAA: an increasingly recognised cause of cognitive impairment

Recent research has expanded understanding of the relationship of CAA with cognitive impairment and highlighted effects that are separate from those due to AD pathology and macrohaemorrhage. A distinct cognitive profile of CAA has been described in living people with CAA-related syndromes, and clinical imaging studies have begun to unravel the mechanisms by which CAA impairs cognition.

Autopsy studies in community-dwelling elderly show that CAA pathology is very common in older people, with moderate to severe accumulation (present in about a third of aged individuals) associated with impaired cognition and adding to the likelihood of dementia even after controlling for the effects of AD and other pathologies.^89–92^ A detailed clinical-pathological study showed that moderate and severe CAA is also independently associated with accelerated decline in global cognition, and specifically processing speed, language skills and episodic memory.^89^ Most recently, two studies report on the neuropsychological profile of CAA in living people with CAA-related clinical syndromes (signifying severe CAA pathology).^93 94^ Both studies excluded patients with dementia, and mostly enrolled patients after CAA-related ICH. Results were essentially concordant, with prominently impaired processing speed observed in both^93 94^ and a high prevalence of mild cognitive impairment (79%).^93^ These differ from the previously mentioned autopsy studies, in which deficits in memory and language were also involved; this may be a reflection of the absence of dementia in these patient groups. The cognitive profile of CAA in these hospital-based studies differed from the community autopsy studies in that episodic memory, the *sine qua non* of AD (and mild cognitive impairment due to AD), was relatively preserved.^93^ Finally, community-based studies have shown associations between strictly lobar microbleeds and impairments in global cognition and visuospatial executive function.^95 96^ Overall, these studies show that cognitive impairment is common in people with CAA, with a profile that may be typical for vascular cognitive impairment (especially prior to developing dementia) but also includes a broader range of impairment that results in a profile that overlaps with that of other diseases.

The prospective association of CAA with risk for dementia has been further demonstrated by the findings of two longitudinal cohort studies of the risk for dementia following ICH. In these prospective cohorts, the incidence of new-onset dementia reached 29% (95% CI 23% to 35%) 4.5 years after ICH onset.^45^ In line with data on pre-existing dementia in patients with ICH,^97^ the risk of incident dementia among non-demented survivors of spontaneous ICH was substantial and higher in patients with lobar ICH. While ICH characteristics like size and location were likely to influence the risk of developing dementia soon after ICH,^98^ predictors of delayed dementia were strongly associated with well-known features of CAA: cSS and cerebral microbleeds. The effect of so-called ‘silent’ chronic lesions suggests that cognitive decline occurring after a lobar ICH is the expression of an underlying subtle small vessel-related process rather than the sole consequence of acute macro-bleeding. Therefore, to prevent cognitive decline in patients with CAA, future studies might focus on modifying and monitoring the appearance of chronic lesions such as cSS or cerebral microbleeds, rather than just trying to avoid macrohaemorrhage. The coexisting issues of haemorrhagic lesions and cognition highlight the need for closer collaborations between stroke centres and memory centres in the field of CAA.

Neuroimaging biomarker studies are beginning to identify correlates of cognitive impairment in the absence of new ICH, implying additional mechanisms of clinically relevant brain injury in CAA. Previous studies showed that cognitive impairment in CAA was associated with higher burden WMH of presumed vascular origin^99 100^ and brain atrophy.^94^ Cortical atrophy may result from CAA-related microinfarction that disconnects white matter tracts,^101^ suggesting that ischaemic injury contributes to cognitive impairments.^102^ The global burden of white matter disconnection is demonstrated by network analyses derived from diffusion-tensor MRI and graph theory methods that consider the brain to be an interconnected network. A study of 38 non-demented patients with CAA showed that global network efficiency was decreased compared with controls, and that among patients with CAA lower global network efficiency explained 34% of the variance in processing speed and 29% of the variance in executive function, without explaining variance in memory.^103^ Over a mean follow-up of 1.3 years, declines in global network efficiency were detectable, particularly for posterior brain network connections, and were associated with declining executive function.^104^

While the associations between cognitive impairment and dementia in CAA (both with and without AD, and in the context of ICH) are increasingly recognised, case reports suggest that CAA can present with other behavioural and psychiatric symptoms, including delirium, depression and personality change.^105 106^ Given the small number of reported cases, it is not clear whether CAA is causative in these cases, or simply a coincident finding. Further data on the mechanisms behind these presentations, as well as their absolute incidence, are needed.

### Impact and interaction of CAA in AD

As well as contributing to cognitive impairment in patients with ICH, CAA might have an analogous independent impact in AD,^89^ which is of particular significance because AD and CAA frequently coexist.^89 90^ Recent neuropathological work has demonstrated that CAA makes an independent cognitive contribution to AD dementia, even after adjusting for other age-related pathologies including AD pathology.^89^ There is evidence that patients with familial AD develop WMH up to 6 years before estimated symptom onset and that this may be a ‘core feature’ of familial AD^107^; the predominantly parietal and occipital distribution of these WMH are consistent with the distribution seen in sporadic CAA.^107 108^ Moreover, cortical atrophy, an imaging finding previously felt to be primarily representative of AD pathology, occurs in CAA even in the absence of coexistent AD pathology^14^; furthermore,  those with strictly lobar microbleeds and AD demonstrate more grey matter atrophy and greater reductions in glucose metabolism than those without.^109^ These findings together suggest that certain clinical and radiological features that have previously been thought to exclusively represent AD pathology may in fact be manifestations of CAA; as a consequence, future treatment strategies for AD that do not consider the impact of CAA may be less efficacious in ameliorating cognitive symptoms.^89^

The apparent impact of CAA in those with AD as well as those with ICH raises questions about the validity of describing CAA as either predominantly ‘haemorrhagic’ or ‘non-haemorrhagic’. Of these subtypes, the former has been associated with the ApoE ε2 allele and haemorrhagic imaging markers of CAA including lobar microbleeds and cSS.^110^ Non-haemorrhagic CAA is associated with the presence of ApoE ε4^110^; ApoE ε4 is also associated with capillary-level CAA as well as AD.^111^ Although it is tempting to consider the latter as a ‘cognitive’ CAA given these associations, evidence suggests that the situation is more complex than this. The presence of capillary CAA does not appear to be associated with cognitive decline,^89^ and although both ApoE ε2 and ApoE ε4 are associated with CAA, it is ApoE ε4 that is associated with more severe CAA on neuropathological examination (as defined by Vonsattel grading).^111 112^ Additionally, ApoE ε4 has been shown to be associated with cSS in a memory clinic population.^41^ Further data on the cognitive impact of ApoE ε2 in CAA, in particular whether it has an effect on cognition beyond the direct damage caused by macrohaemorrhage, are needed.

## Management and treatment dilemmas

### Transient focal neurological episodes

**Figure 3:**
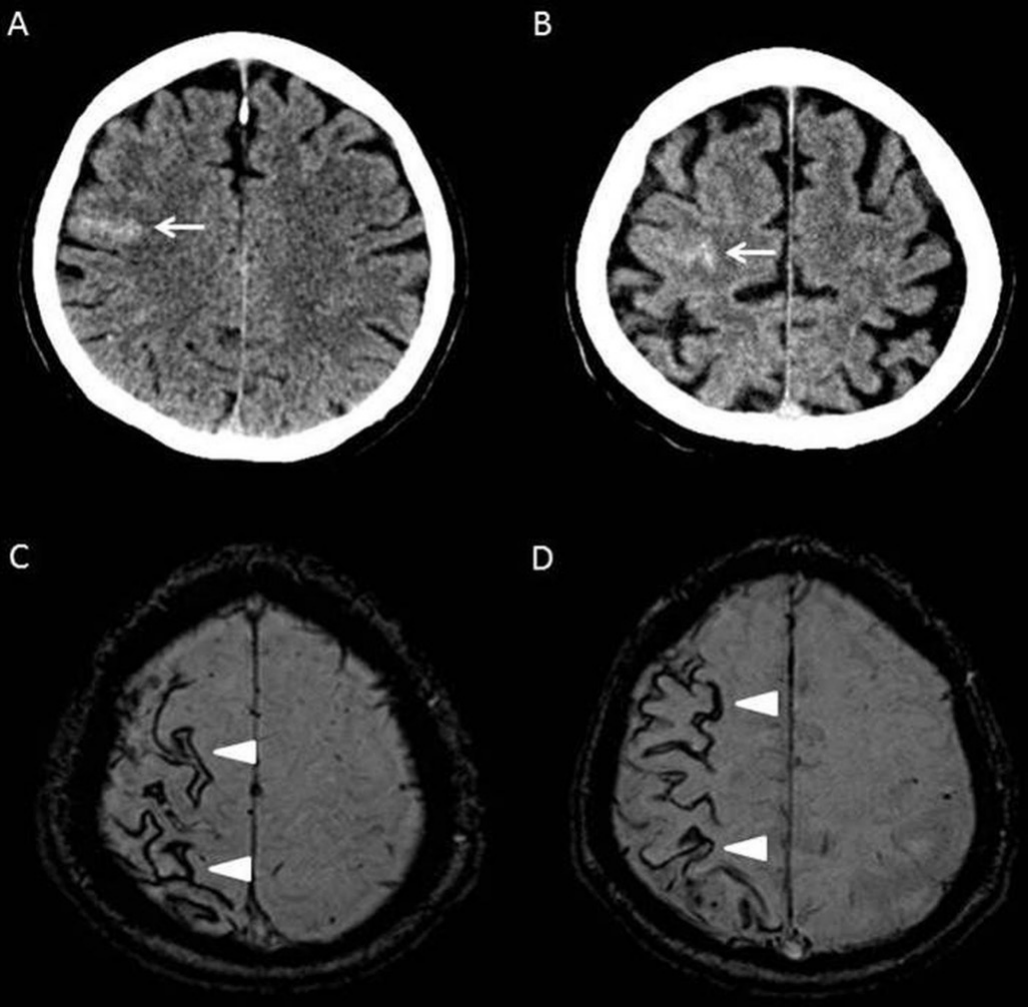
Imaging findings in CAA-associated TFNE. Images from a 76-year-old patient who presented with migratory left-sided sensory symptoms consistent with CAA-associated TFNE. His original CT (A) shows a hyperdense area in keeping with an acute cSAH (arrow). Three months later he had a similar episode; repeat CT (B) at this time demonstrated another acute cSAH nearby (arrow). Subsequent susceptibility weighted MRI (C and D) showed widespread disseminated cSS affecting the right hemisphere (arrowheads). CAA, cerebral amyloid angiopathy; cSAH, convexity subarachnoid haemorrhage; cSS, cortical superficial siderosis; TFNE, transient focal neurological episodes.

The last 5 years have seen a continued development of our understanding and recognition of TFNEs, which are established as a common clinical feature of CAA. There is a greater appreciation of the range of symptoms with which TFNE can present, in particular ‘negative’ or ‘transient ischaemic attack (TIA)-like’ symptoms such as motor weakness, dysphasia or visual loss, which may be present in up to half of all TFNE presentations.^113^ Spreading onset and recurrent stereotyped attacks seem to be typical.^113^ There is increasing evidence that TFNE frequently occurs as a result of acute cSAH,^113–116^ with symptoms correlated with the site of cSAH.^114^ This acute cSAH has been shown to evolve (often over weeks to months^44^) by deposition of haemosiderin in superficial cortical layers as cSS, a recognised MRI marker of CAA (figure 3).^32 114 115^ TFNE seems to be associated with focal cSS in particular.^32^ TFNE and cSAH both also appear to be associated with an increased risk of subsequent ICH.^35 113 115^ Rather than a direct consequence of the individual site of cSAH or cSS, the increased ICH risk could be because TFNE (via their association with cSAH and cSS) is a marker of overall leptomeningeal CAA severity; indeed, there is some evidence that future haemorrhages tend to occur in locations distant from sites of cSAH or cSS.^114^ The mechanism by which an acute cSAH results in TFNE remains unknown. Electroencephalography (EEG) changes, namely intermittent focal slowing, have been demonstrated in a subset of patients with TFNE^114^; this is of interest as similar EEG findings have been associated with the cortical spreading depression believed to underlie migraine aura.^113 114^ Cortical spreading depression has also been observed in patients with aneurysmal SAH, and is believed to contribute to the delayed ischaemic damage seen in these patients.^117–119^ However, EEG changes were only present in a small number of patients with TFNE, and it is not clear whether all TFNE presentations (for example, limb-jerking) can be explained by cortical spreading depression alone; seizure activity might play a role in some patients. Although there is increasing anecdotal data that antiepileptic and antimigraine medications (such as levetiracetam or topiramate) might reduce the frequency or severity of TFNE symptoms,^44 113 116^ they may also remit spontaneously; controlled trial data are lacking. Recognition of CAA-related TFNE (by assessing the clinical features and performing appropriate acute neuroimaging) is critical to avoid potentially hazardous exposure to antithrombotic drugs, the standard treatment for TIAs.

### Management of patients with CAA with indications for antithrombotics or anticoagulants

The thorniest dilemmas in management of patients with CAA occur when there is an unrelated indication for antithrombotic therapy. Unfortunately, this is all too common because evidence-based indications for antithrombotic therapy, such as atrial fibrillation or vaso-occlusive disease, accrue with age, just as CAA does. A study of lobar ICH survivors showed that despite an episode of life-threatening bleeding, more than 20% had antithrombotics initiated after hospital discharge.^120^

With an average ICH recurrence rate in CAA of about 9% per year,^121^ antithrombotic strategies that increase the relative risk of ICH by more than about 50% are likely to outweigh any antithrombotic-associated reduced risk of thrombosis, even in patients with atrial fibrillation, probably causing more harm than good. Much of the early data for restarting oral anticoagulation (OAC) after an OAC-related ICH came from small hospital studies with inconclusive results.^122–125^ Three larger registry studies^126–128^ have since demonstrated that restarting OAC after an OAC-related ICH might be of benefit, with a clear reduction in the risk of ischaemic stroke in all cases. With regard to recurrent ICH risk, the results are more indeterminate, with one study showing a slight risk reduction,^126^ another no increase in risk^127^ and another showing an increased risk.^128^ A recent meta-analysis^129^ including 5306 patients with anticoagulation-associated ICH from eight studies (nearly all evaluating treatment with vitamin K antagonists) suggested that restarting anticoagulation was associated with a lower risk of thromboembolic complications (pooled relative risk, 0.34; 95% CI 0.25 to 0.45; Q=5.12, p for heterogeneity=0.28), with no increased risk of recurrent ICH (although with significant heterogeneity; pooled relative risk, 1.01; 95% CI 0.58 to 1.77; Q=24.68, p for heterogeneity <0.001). There are also some data^126 130 131^ that restarting anticoagulation in those with previous ICH and atrial fibrillation is associated with reduced mortality. Unfortunately, none of these observational studies distinguish between CAA-related and non-CAA-related ICH; given the recurrent ICH risk is higher in those with CAA,^17 121 132^ it is difficult to know whether the above findings are applicable to these patients. Additionally, these studies are retrospective and observational, thus lacking randomised comparisons, and may be prone to further inherent physician treatment biases about which patients with ICH are chosen to resume anticoagulation and which are not. Currently we suggest that it is probably not indicated to use warfarin anticoagulation in patients with CAA and a history of ICH,^133^ as warfarin reinitiation may increase the risk of recurrent ICH by more than fivefold in ICH survivors.^123^ The risk for new symptomatic haemorrhage in patients with CAA-related TFNE or cSAH is not as well defined but probably also substantial and similar to that after CAA-ICH^35 113^; therefore, warfarin anticoagulation should probably be avoided in these patients as well.

The use of aspirin is an alternate strategy for patients with atrial fibrillation and CAA-related ICH, but has limited evidence of efficacy for atrial fibrillation-related stroke prevention.^134^ The non-vitamin K antagonist oral anticoagulants (NOACs) have proven efficacy in atrial fibrillation-related stroke prevention but only half the risk of intracranial haemorrhage as warfarin,^135^ and may thus be a valuable alternative to warfarin in ICH survivors. In the only randomised comparison of a NOAC with aspirin, in patients considered unsuitable for warfarin, apixaban was more effective at preventing thrombotic events with no difference in rates of overall bleeding or intracranial bleeding.^136^ In an MRI substudy of AVERROES (Apixaban Versus Acetylsalicylic Acid [ to Prevent Stroke in Atrial Fibrillation Patients Who Have Failed or Are Unsuitable for Vitamin K Antagonist Treatment),^137^ the number of new microbleeds was also not different between patients assigned to apixaban versus aspirin. Finally, the use of devices to occlude the left atrial appendage could be considered in eligible patients with atrial fibrillation and history of ICH,^133^ although the optimal postimplantation antithrombotic strategy has not been defined in prospective studies in patients with CAA. Further randomised trials of anticoagulation after ICH are clearly needed and are underway.^138^

The risks of antithrombotic therapies in patients with incidentally discovered lobar microbleeds, possibly representing asymptomatic CAA, have been much debated^139^ but with little evidence on which to base decisions. In the general population, participants with lobar microbleeds have a very low annual risk of ICH (only 0.6% over mean 4.9 years of follow-up in one study^140^); however, the degree to which this risk would be increased by anticoagulation is uncertain. In patients with ischaemic stroke or TIA, cerebral microbleeds increase the risk of both future ischaemic stroke and ICH; the absolute risk of ischaemic stroke exceeds that of ICH, but in those with more than five microbleeds (mainly treated with antiplatelet therapy) these risks seem to be finely balanced.^141^ A scientific statement from the American Heart Association^142^ suggests that routine guideline recommended care for atrial fibrillation should be unchanged even when lobar microbleeds are present, with a preference for NOACs when anticoagulation is indicated. MRI screening prior to initiating anticoagulation is of unknown value and unlikely to be cost-effective.^143^ Ongoing cohort studies in patients with atrial fibrillation, ischaemic stroke and MRI, treated with OAC, will provide better prospective information on microbleeds and future ICH risk in this population.^144 145^

### Statins

Statin use in patients with, or at risk of, ICH has been controversial since the SPARCL (Stroke Prevention by Aggressive Reduction in Cholesterol Levels) trial in 4731 patients with recent stroke (due to ischaemia or haemorrhage) or TIA found an increased risk of recurrent ICH in those taking high-dose atorvastatin (55 ICH vs 33 in the placebo group; hazard ratio, HR, 1.68, 95% CI 1.09 to 2.58).^146^ In multivariable Cox regression, ICH risk was sixfold higher in those having a haemorrhagic stroke as the entry event (HR 5.65, 95% CI 2.82 to 11.30, p<0.001),^146^ and the risk of ICH was highest in those with SVD (HR 4.99, 95% CI 1.71 to 14.61).^146 147^ The only other randomised study to consider the effect of statins in patients with stroke (ischaemic stroke) was the Heart Protection Study (HPS)^148^; a meta-analysis combining data from SPARCL and HPS found that while statin use was protective for ischaemic stroke (relative risk, RR, 0.80, 95% CI 0.78 to 0.99), the risk for ICH was increased (RR 1.73, 95% CI 1.19 to 2.50).^149^

While the data for statins and ICH in general remain conflicting,^150–159^ observational data suggest that statins might be particularly associated with ICH in the context of CAA. A trend for association between statin use and lobar ICH for those with the ApoE *ε4/ε4* or ApoE *ε2/ε4* genotypes has been described,^160^ and the more recent Multicenter Study on Cerebral Haemorrhage in Italy (MUCH-Italy) study^161^ reported that the greatest impact of statins on future ICH risk appears to be in those with lobar haemorrhages. A decision analysis suggested that statin use in survivors of lobar ICH was associated with a reduction in quality-adjusted life years.^162^ There is also evidence for an association between statin use and the presence of lobar microbleeds.^163 164^ Despite the relative paucity of data in this area and its clear limitations (most obviously the use of lobar macrohaemorrhages and microhaemorrhages as surrogate markers for CAA, and the absence of information on the influence of statin dose), recent decision analyses support caution when prescribing statins in those with CAA.^151 162^ Data from randomised prospective studies including ICH survivors are needed to improve our understanding of the possible risks of statin use in CAA.

### Immunotherapy

June 2013 saw the start of the first therapeutic clinical trial in CAA, using the humanised monoclonal antibody ponezumab.^165 166^ The antibody, initially trialled in AD,^166–168^ is of interest to the CAA community given its specificity for Aβ40, the predominant Aβ subtype in vascular deposits,^3 166^ and its promising results in animal models.^169^ There were questions about which outcome markers to use for this relatively short trial,^31^ as well as significant safety concerns given the common occurrence of ARIA following Aβ immunotherapy in AD.^82^ Given that ARIA is pathologically characterised by a shift of Aβ into the vasculature and the development of imaging features consistent with CAA,^5 85^ current recommendations advise excluding patients with high microbleed counts from AD immunotherapy trials.^170^ However, despite worries that immunotherapy in CAA could lead to clinical deteriorations in these patients, ponezumab was well tolerated, with no ARIA-like events (Claire Leurent, Pfizer, personal communication 2017). The formal results of this study, which used occipital vascular reactivity to visual stimulation as a primary outcome measure,^165^ are expected later this year.

## What’s next for CAA?

In the last 5 years we have seen real advances in our understanding of CAA, and there is excitement about potential future progress. The planned revision of the current Boston criteria to better reflect recent clinical and radiological discoveries is underway, and it is expected that any new criteria will address the relevance of non-haemorrhagic markers (for example, perivascular spaces in the centrum semiovale), whether the presence of deep microbleeds truly excludes a diagnosis of CAA, and whether different diagnostic criteria are needed for its haemorrhagic and cognitive subtypes. This should improve our ability to define disease presence, severity and subtypes, which might help guide clinical decision making and future trials with regard to antithrombotic, anticoagulant and statin use.

Developing treatments for CAA remains a priority; the suggestion that immunotherapy in CAA might be safe opens the road for the development of new agents with a similar mode of action. Other strategies, for example the drug (R)-1-[6-[(R)-2-carboxy-pyrrolidin-1-yl]-6-oxo-hexanoyl]pyrrolidine-2-carboxylic acid (CPHPC), which targets serum amyloid P component, also hold promise.^171^ However, while the hope is that therapies that target vascular amyloid will be successful, there is a risk that without significant progress in our knowledge of how and why CAA develops, identifying an effective treatment will be impossible. For this reason, advances in our understanding of CAA pathophysiology and how this translates into its clinical and radiological manifestations are a crucial development needed to advance the field in future years.

Identifying an effective therapeutic approach for CAA might have a critical bearing on the treatment of AD; given current recommendations excluding those with high microbleed counts from AD immunotherapy trials^170^ and the growing evidence that ARIA is common and possibly a marker of treatment efficacy,^5 83–86^ being able to understand and potentially treat CAA-related effects could improve safety and increase the number of patients with AD eligible for treatment with these agents. This raises the possibility of combination immunotherapy, with parenchymal and vascular Aβ being targeted at different stages of treatment. New evidence on the impact of potentially modifiable risk factors for CAA, in particular blood pressure control, is also needed. While a subgroup analysis of PROGRESS (Perindopril Protection Against Recurrent Stroke Study)^172^ showed that those with probable CAA (although with limited diagnostic evaluation) had a 77% reduction in ICH with blood pressure lowering, there is still no guidance on specific blood pressure targets or how aggressively this should be managed in patients without macrohaemorrhage. As well as preventing recurrent ICH, there is interest in whether blood pressure has an impact on the progression and burden of CAA, in a manner analogous to that observed in age-related deep-perforating arteriopathy, another common cerebral SVD.^173 174^ Further randomised controlled trials, including the use of neuroimaging surrogate markers of CAA, will be important in determining how blood pressure control (of both level and variability) might influence the natural history of CAA. Other important areas of research include the natural history of CAA (both with and without macrohaemorrhage), identifying biomarkers for early disease and the nature and extent of cognitive impact of CAA in ICH and AD.

## Conclusions

The clinical impact of CAA is no longer disputed, and the last 5 years have witnessed a rapid increase in new knowledge in this field. Newly identified biomarkers are improving our diagnostic capability and mechanistic understanding, as well as providing practical outcome markers in clinical trials. The interaction between CAA and AD remains of great interest, both in terms of pathological and cognitive implications, and may be key to the successful use of immunotherapies in AD and CAA in future. The next 5 years promise new and exciting advances with the prospect of earlier, more accurate diagnosis and ultimately rational preventive and disease-modifying treatments.
